# Electron‐Hole Separation Dynamics and Optoelectronic Properties of a PCE10:FOIC Blend

**DOI:** 10.1002/smll.202505063

**Published:** 2025-07-02

**Authors:** Giuseppe Ammirati, Stefano Turchini, Francesco Toschi, Patrick O'Keeffe, Alessandra Paladini, Giuseppe Mattioli, Paolo Moras, Polina M. Sheverdyaeva, Valeria Milotti, Christoph J. Brabec, Michael Wagner, Iain McCulloch, Aldo Di Carlo, Daniele Catone

**Affiliations:** ^1^ CNR‐Istituto di Struttura della Materia (CNR‐ISM) EuroFEL Support Laboratory (EFSL) Via del Fosso del Cavaliere 100 Rome 00133 Italy; ^2^ CNR‐Istituto di Struttura della Materia (CNR‐ISM) Monterotondo Scalo 00015 Italy; ^3^ CNR‐Istituto di Struttura della Materia (CNR‐ISM) SS 14, Km 163.5 Trieste I‐34149 Italy; ^4^ Dept. of High Throughput Methods in Photovoltaics Forschungszentrum Jülich GmbH Helmholtz Institute Erlangen‐Nürnberg for Renewable Energy (HI‐ERN) 91058 Erlangen Germany; ^5^ Friedrich‐Alexander‐Universität Erlangen‐Nürnberg Materials for Electronics and Energy Technology (i‐MEET) Erlangen Germany; ^6^ Andlinger Center for Energy and the Environment Department of Electrical and Computer Engineering Princeton University Princeton NJ 08544 USA; ^7^ Department of Chemistry Chemistry Research Laboratory Oxford University Oxford OX1 3TA UK; ^8^ CHOSE University of Rome “Tor Vergata” Rome 00133 Italy

**Keywords:** band diagram, charge dynamics, electron‐hole separation, organic photovoltaic, photovoltaics

## Abstract

Understanding charge separation dynamics in organic semiconductor blends is crucial for optimizing the performance of organic photovoltaic solar cells. In this study, the optoelectronic properties and charge separation dynamics of a PCE10:FOIC blend, by combining steady‐state and time‐resolved spectroscopies with high‐level DFT calculations. Femtosecond transient absorption spectroscopy revealed a significant reduction of the exciton‐exciton annihilation recombination rate in the acceptor when incorporated into the blend, compared to its pristine form. This reduction is attributed to a decrease in exciton density within the acceptor, driven by an efficient hole‐separation process that is characterized by following the temporal evolution of the transient signals associated with the excited states of the donor when the acceptor is selectively excited within the blend. The analysis of these dynamics enabled the estimation of the hole separation time constant from the acceptor to the donor, yielding a time constant of (1.3 ± 0.3) ps. Additionally, this study allowed the quantification of exciton diffusion and revealed a charge separation efficiency of ≈60%, providing valuable insights for the design of next‐generation organic photovoltaic materials with enhanced charge separation and improved device efficiency.

## Graphical abstract

Charge separation and exciton‐exciton annihilation in PCE10:FOIC blends are revealed through transient absorption spectroscopy and DFT calculations. Selective excitation of FOIC shows a rapid hole transfer (≈1.3 ps) to PCE10, leading to the ≈60% charge separation efficiency‐key for improving organic solar cell performance.

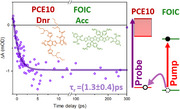

## 1 Introduction

Organic photovoltaic (OPV) solar cells have emerged as a promising avenue for renewable energy production, offering a cost‐effective alternative to traditional silicon‐based solar cells. Their qualities, including flexibility, lightness, semi‐transparency, and ease of processing, make them attractive for various applications such as outdoor and building‐integrated photovoltaics, and wearable electronic devices.^[^ ^1^, ^2^, ^3^, ^4^ ^]^ To further improve the performance of such cells, a comprehensive understanding of the intricate mechanisms governing the photovoltaic process is highly desirable. In bulk heterojunction (BHJ) architectures, excitons typically dissociate at the donor‐acceptor (D‐A) interface through charge separation (CS) processes, leading to the formation of free charges that are subsequently extracted as photocurrent.^[^ ^5^, ^6^, ^7^ ^]^ The energy offset between donor and acceptor affects the open‐circuit voltage (V_oc_); a small energy level offset is preferred to maximize the V_oc_, but this, on the other hand, can induce slower CS rates and increased exciton recombination, potentially reducing current generation.^[^ ^8^, ^9^ ^]^ Thus, achieving an optimal balance in the energy offset is crucial for enhancing the efficiencies in OPV systems. Fullerene‐based literature has suggested that a minimum level offset of at least 0.3 eV is necessary for efficient CS.^[^ ^8^, ^10^, ^11^, ^12^, ^13^ ^]^ The introduction of nonfullerene acceptors (NFAs) into BHJ structures has recently led to a significant performance boost,^[^ ^14^, ^15^, ^16^ ^]^ achieving record efficiencies close to 20%.^[^ ^17^ ^]^ This success is attributed to their high absorption in the near‐infrared region, even with minimal energy offset. Recent findings have shown that when the highest occupied molecular orbital (HOMO) offset drops below 0.2 eV, the CS rate decreases. This leads to a lower short‐circuit current, which overcompensates for the V_oc_ gain.

Femtosecond Transient Absorption Spectroscopy (FTAS) is a powerful tool for investigating photophysical processes occurring in the active materials for OPV applications. This makes it particularly relevant for studying recombination dynamics and charge transfer at D‐A bulk heterojunctions.^[^ ^12^, ^18^, ^19^, ^20^, ^21^, ^22^, ^23^, ^24^ ^]^ By offering a quantitative and time‐resolved analysis of charge generation, transfer, and recombination dynamics, FTAS provides critical insights that drive advancements in the understanding and optimization of OPV materials, ultimately contributing to enhanced device performance. Remarkably, by enabling the direct observation of transient species such as singlet and triplet excitons, charge transfer and CS states, and free carriers, FTAS allows the identification and characterization of excitonic and charge‐related processes, crucial for understanding the dissociation of excitons at the D‐A interface and the generation of free carriers. By resolving the temporal evolution of these species, FTAS distinguishes between geminate recombination of tightly bound electron‐hole pairs^[^ ^19^, ^25^, ^26^, ^27^ ^]^ and bimolecular recombination of independently generated charges.^[^ ^28^ ^]^ Furthermore, its ability to monitor processes across a broad timescale, from femtoseconds to microseconds, creates a detailed analysis of both ultrafast charge transfer events^[^ ^29^, ^30^, ^31^, ^32^, ^33^ ^]^ and longer‐lived recombination pathways.^[^ ^34^ ^]^

In this context, the ultrafast separation dynamics of both electrons and holes in BHJs were investigated with different configurations, demonstrating that the intrinsic hole separation remains on the picosecond time scale, even for a near‐zero driving force.^[^ ^12^, ^32^ ^]^ However, the crucial connection between recombination losses and CS processes is missing. Whether recombination occurs geminately, where an electron and a hole recombine without ever leaving their point of origin, or through intermolecular interactions, it ultimately leads to a decrease in power conversion efficiency.^[^ ^35^ ^]^ In the context of D‐A systems, FTAS also serves as a diagnostic tool for assigning spectral features to specific components, allowing the evaluation of the component‐dependent efficiency of CS and charge collection. This capability is essential for optimizing the design of bulk heterojunctions and improving the understanding of energy losses that limit device performance.

In this work, we employ a combination of advanced experimental techniques, assisted by atomistic simulations, to study the optoelectronic properties and CS dynamics of a D‐A blend composed of PCE10 (also known as PTB7‐Th) as D and FOIC as A. Such a blend is characterized by a high transparency in the visible spectrum and by promising power conversion efficiencies, making it ideal for use as active material in semitransparent solar cells.^[^ ^18^, ^36^, ^37^, ^38^ ^]^ Through steady‐state and time‐resolved spectroscopic techniques, supported by high‐level DFT calculations, we provide an in‐depth analysis of absorption spectra, band diagrams, and excited‐state properties of one‐component thin films as well as blends, enabling us to explore the exciton‐exciton annihilation dynamics and the hole separation mechanism between the A and D within the blend.

## 2 Results and Discussion

### 2.1 Steady‐State Optical and Electronic Properties

**Figure 1:**
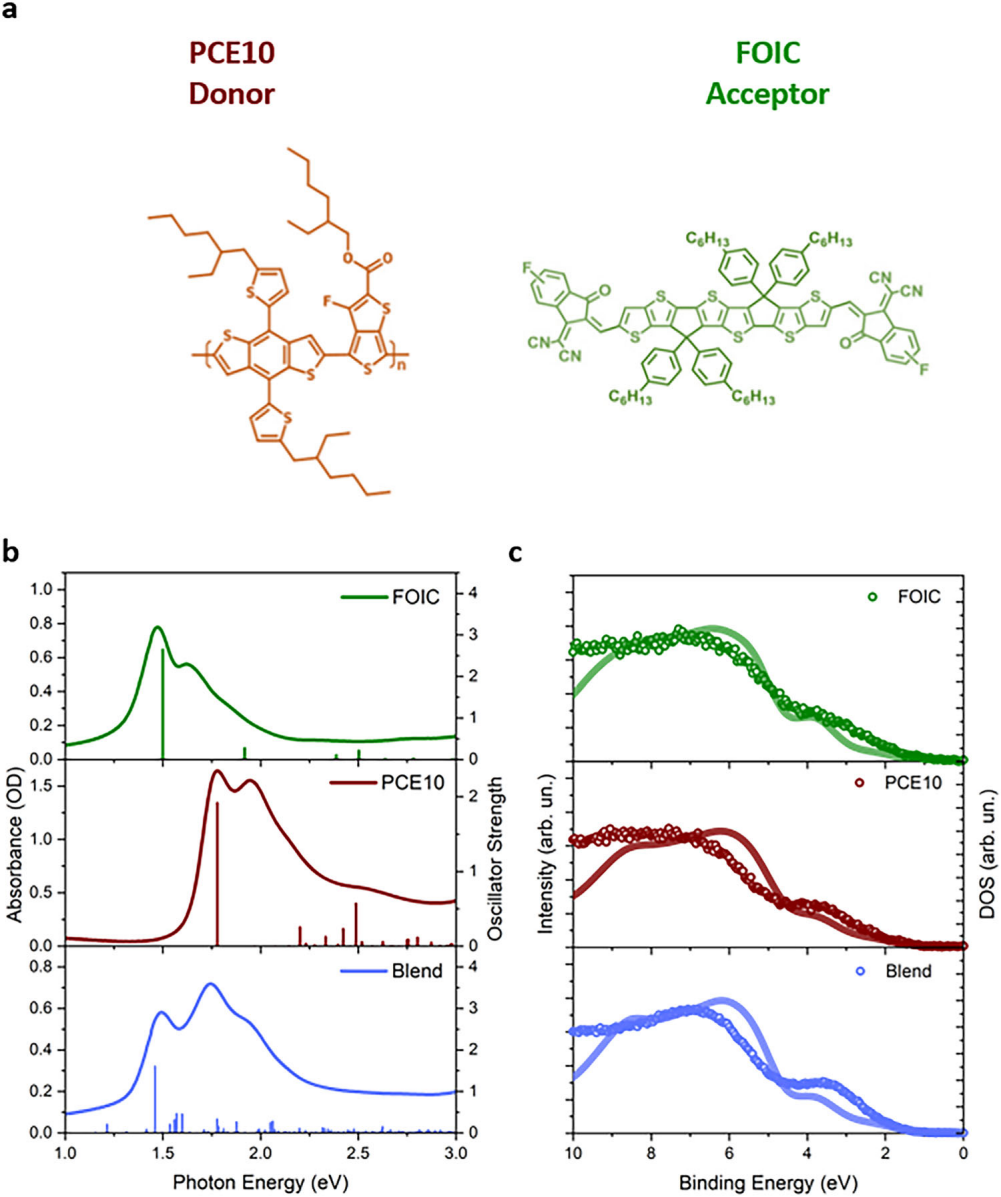
a) Molecular structure of PCE10 (D) and FOIC (A); b) experimental (line) and theoretical (bars) absorption spectra of FOIC (green), PCE10 (red), and Blend (blue) together with the spectra profiles of PCE10 (D) and FOIC (A). The theoretical results of PCE10 were rigidly shifted by +390 meV to align them with the experiment. c) Experimental photoelectron spectra of the film of FOIC (green scatter), PCE10 (orange scatter), and Blend (blue scatter) acquired at the photon energy of 240 eV along with the corresponding theoretical DOS (lines).

The molecular structures of FOIC (A) and PCE10 (D) are shown in **Figure** **1**a. The absorption spectra of FOIC (A), PCE10 (D), and PCE10:FOIC (Blend), acquired with a PerkinElmer Lambda19 spectrophotometer, are shown in Figure 1b together with the optical transitions predicted by DFT and reported as bar diagrams (see Section S1 of Supporting Information for further details). FOIC shows a broad absorption peak at 1.45 eV with additional structures at 1.7 and 1.8 eV. PCE10 exhibits a broad absorption feature with structures at 1.72, 2.0, 2.2, and 2.5 eV. The absorption spectrum of the Blend shows features that can be attributed to both A and D contributions. In particular, it is possible to assign the peak at 1.52 eV to a main A contribution, although slightly blue‐shifted with respect to the bare FOIC, while the shoulders at ≈1.7 and 2.0 eV resemble the D spectral features. Furthermore, the tails at higher energies in the experimental spectra are likely attributed to vibrational states of the molecular systems, an aspect not taken into account by the calculations. In the case of PCE10, the simulations give the first absorption peak at 1.39 eV for the tetramer (see Table S1, Supporting Information), which is significantly redshifted compared to the experimental peak at 1.72 eV. Thus, the theoretical results obtained for PCE10 and shown in Figure 1b are rigidly shifted by +390 meV to align them with the experimental spectrum. This discrepancy likely arises from aggregation effects between polymer chains, not implemented in our simulations, which lead to an underestimation of the calculated absorption energy features. The optical band gaps (E_g_) for the studied materials were estimated from the acquired absorption spectra, resulting in: 1.26 eV for FOIC; 1.58 eV for PCE10; 1.32 eV for Blend (see Section S2 of Supporting Information for further details).

**Figure 2:**
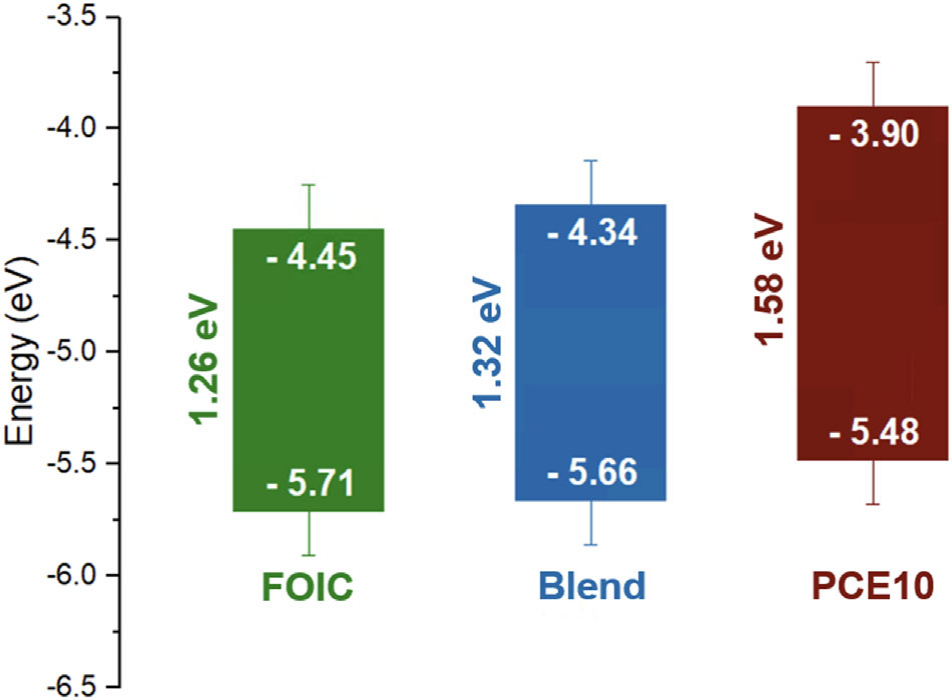
Experimental band diagram of FOIC, Blend, and PCE10. The energy of the valence band was evaluated by the onset of the PES spectra obtained at 75 eV, while the energy of the conduction band was then calculated by adding to the valence band the E_g_ obtained from the absorption spectra. The energy values reported were estimated with respect to the vacuum energy.

Figure 1c shows the PES spectra in the Valence Band (VB) region, obtained with a photon energy of 240 eV for FOIC, PCE10 and Blend (scattered point) together with the calculated DOS (solid lines), generated by the convolution of B3LYP Kohn‐Sham eigenvalues with Gaussian functions (σ = 32 meV) and shifted by +3.0 eV to match the experimental data. The theoretical predictions are in satisfactory agreement with the experimental data. The estimation of the binding energy onset was used to evaluate the VB energy with respect to the vacuum energy of the studied materials: 1.07 eV for FOIC; 0.97 eV for PCE10; 1.15 eV for Blend. The energy of the Conduction Band (CB) was then calculated by adding to the VB the E_g_ obtained from the absorption spectra. In this way, the band diagrams reported in **Figure** **2** (for the details, see Section S2 of the Supporting Information) were generated.

These results, which are in good agreement with the theoretical band diagram shown in Figure S1, allowed us to estimate the energy offset between the lowest unoccupied molecular orbital (LUMO) of D and the HOMO of A, which is an important parameter that strongly influences the optoelectronic properties of these materials and their performance when used for solar cell devices. Based on the experimental band diagram, the energy difference between the PCE10 VB maximum (−5.48 eV) and the FOIC CB minimum (−4.45 eV) yields an offset of 1.03 eV. Additionally, the HOMO offset and LUMO offset, determined by the energy difference between FOIC and PCE10, are 0.23 eV and 0.55 eV, respectively.^[^ ^39^ ^]^ These results are consistent with other NFAs developed for photovoltaic applications, which have demonstrated good performance^[^ ^40^ ^]^ with reduced losses that positively impact V_oc_ and fill factor.^[^ ^41^ ^]^

### 2.2 Transient Absorption Spectroscopy and Ultrafast Dynamics in FOIC, PCE10, and Blend

**Figure 3:**
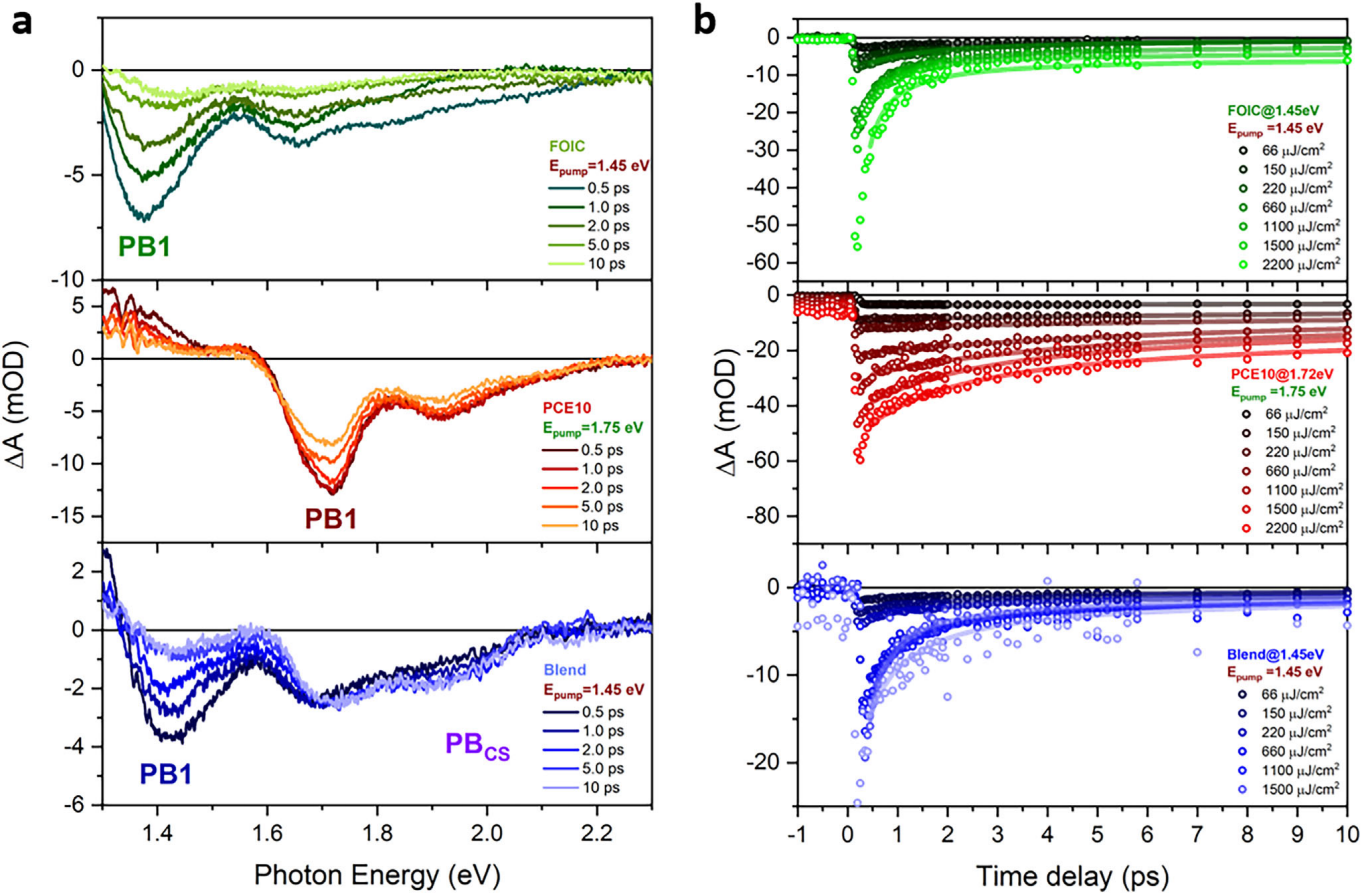
a) TA spectra obtained at the pump fluence of 220 µJ cm^−2^ for FOIC (green lines) at the pump photon energy of 1.45 eV, PCE10 (red lines) at the pump photon energy of 1.75 eV, and Blend (blue lines) at the pump photon energy of 1.45 eV. b) Experimental (scatter) and fit (line) of the temporal dynamics of PB1 signals at selected fluences for FOIC (green), PCE10 (red), and Blend (blue) in the same experimental conditions. The non‐zero signals at negative time delays are due to pump scattering.

On the basis of the ground‐state electronic structure of FOIC, PCE10, and Blend reported above, it is possible to discuss the results obtained from the FTAS. **Figure** **3**a shows the transient absorption (TA) spectra of FOIC and Blend obtained with a pump at 1.45 eV and a fluence of 220 µJ cm^−2^, and the TA spectrum of PCE10 acquired with a pump at 1.75 eV. All the TA spectra show photobleaching (PB) signals (negative signals) that correspond to the peaks present in the absorption spectra. We observed a distinct behavior in the Blend when excited by a pump at 1.75 eV (see Figure S4 in Supporting Information) and at 1.45 eV. When excited at 1.75 eV, the pump energy is sufficient to excite both the A and D components in the Blend, resulting in PB signals that resemble features in the absorption spectrum. In contrast, when excited at 1.45 eV, the pump energy can only excite the A component dispersed in the Blend (see spectra in Figure 1a), leading to TA spectra that closely resemble those of the pure FOIC. Additionally, the broad PB signal at probe energies ≈1.9 eV, corresponding to the D contributions, shows a slower rise compared to other lower‐energy PB signals. The nature and the dynamics of this PB feature will be discussed in detail later in the text.

In this context, we have studied the time dependence of the TA signals as a function of the excitation fluence, and consequently, as the exciton density increases. Figure 3b shows the decay dynamics of bleaching signals, labeled as PB1, for FOIC, PCE10 and Blend at selected excitation fluences for pump energies at 1.75 eV for PCE10 and 1.45 eV for FOIC and Blend (for the TA spectra obtained in other excitation conditions, see Section S3 in the Supporting Information). The data show a decay trend in the first picoseconds, which becomes faster as the exciton density increases, suggesting that the dynamics is influenced by a scattering process. This trend was assigned to an exciton‐exciton annihilation (EEA) process, i.e., a nonradiative many‐body interaction that involves the energy transfer from one exciton to another.^[^ ^42^ ^]^ In fact, this recombination process is described as a bimolecular process and was established as the dominant recombination mechanism within organic materials and in systems with reduced dimensionality^[^ ^43^, ^44^, ^45^, ^46^ ^]^ (see Section S2 in the Supporting Information for further details on the data fitting procedure).

Although the exciton density used in this work is higher than that of OPV devices under typical operating conditions, where exciton splitting is the main process, estimating the EEA is of significant interest. This rate serves as a valuable tool for evaluating the excitonic diffusion coefficient (D_ex_), a crucial parameter in the development of efficient organic photovoltaic devices. The relationship between EEA and D is as follows:^[^ ^47^ ^]^

(1) $$D_{ex}=\frac{\gamma }{4\pi R}\;$$

where *γ* is the EEA rate constant and *R* is the exciton annihilation radius (approximated to 1 nm, as estimated in both experimental^[^ ^48^ ^]^ and theoretical^[^ ^49^ ^]^ studies).

**Figure 4:**
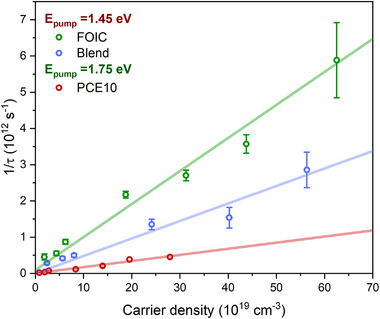
Experimental data (scatter) and fitting curve (line) of the EEA rate for FOIC (green), PCE10 (red), and Blend (blue). The linear fit was used to estimate the EEA rate constant γ for the studied materials. Both the estimated carrier density and the EEA decay time are reported in Table S4 (Supporting Information).

**Figure 5:**
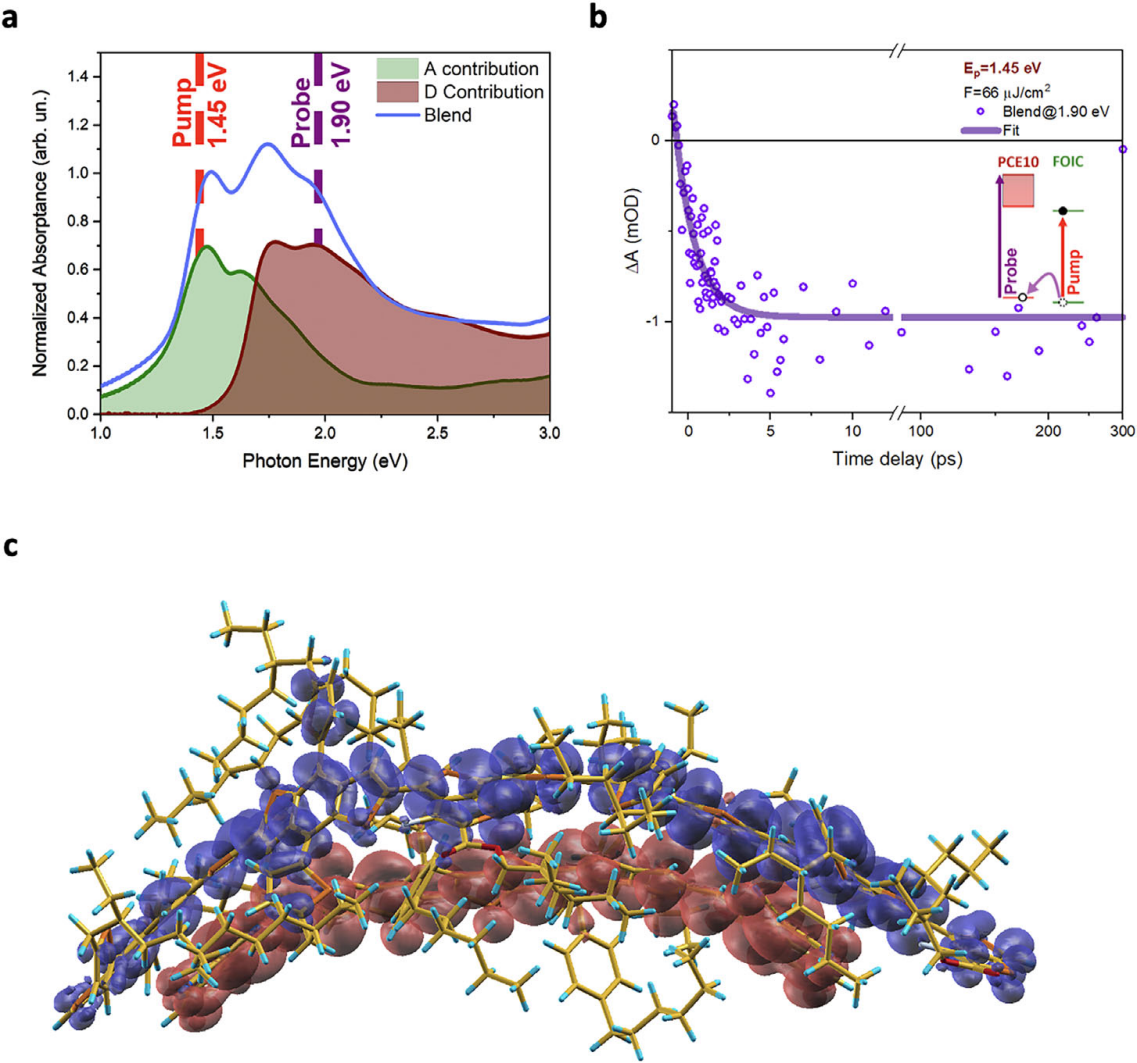
a) Absorption in the Blend (blue line) and the A (green area), and the D (red zone) contribution at the absorption spectrum. b) Temporal dynamics excited at the pump energy of 1.45 eV and the fluence of 66 µJ cm^−2^ at the probe photon energy of 1.90 eV with an energy integration window of ≈±5 meV. Inset: simplified schematization of the hole separation process between D (PCE10) and A (FOIC). c) Density difference between the ground state and the lowest‐energy excited state of a FOIC: PCE10(4) dyad, calculated using TDDFT. Upon excitation, the dyad evolves toward a charge‐transfer state where the photoinduced hole is wholly localized on PCE10(4) (blue regions), whereas the electron is localized on FOIC (red regions).

**Figure** **4** reports the EEA rates for FOIC (green), PCE10 (red), and Blend (blue), together with the corresponding linear fit used to estimate the γ values for the studied materials. The estimated EEA rates and diffusion constant for FOIC, PCE10, and Blend are reported in **Table** **1**, and are comparable to those measured for other organic materials used in photovoltaics.^[^ ^43^, ^50^, ^51^ ^]^ Here we highlight that, by exciting Blend with a pump at 1.45 eV, we are mainly observing the properties of the A (FOIC) molecule when dispersed in the Blend (see **Figure** **5**a).

**Table 1:** EEA rate constant γ and estimated diffusion coefficient D for FOIC, PCE10, and Blend.

	FOIC	PCE10	A [FOIC] in Blend
γ (10^−9^ cm^3 ^ s^−1^)	9.1 ± 0.4	1.7 ± 0.2	4.8 ± 0.4
D_ex_ (10^−3^ cm^2 ^ s^−1^)	7.2	1.4	3.8

The lower γ and the consequent lower D_ex_ exhibited by PCE10 could be attributed to a lesser structural order compared to FOIC.^[^ ^50^ ^]^ Conversely, the lower γ of the A (FOIC) within the Blend compared to pure FOIC indicates how the EEA recombination process is influenced by the interaction with the D (PCE10). Specifically, since EEA is a scattering process, the reduction in its rate constant suggests a decrease in the exciton density of FOIC within the Blend that may be partially due to CS, namely the hole displacement process from A to D. It is worth noting that the EEA rate was not evaluated for the D in the Blend, as it is impossible to excite PCE10 exclusively without also exciting FOIC.

### 2.3 Charge Separation Dynamics in FOIC: PCE10 Blend

To further investigate electron‐hole separation, we analyzed the broad PB signal, labeled as PB_CS_, appearing in the 1.8–2.2 eV energy range a few picoseconds after the excitation of the Blend. This signal is almost entirely attributed to spectral features related to the PCE10 component (see Figure 3a and Figure 5a). This process effectively leads the PCE10 in a CS state, leading to a reduced population of PCE10 in its ground state after the FOIC photoexcitation (see the scheme in Figure 5b). Thus, following the temporal trend of the PB signal at 1.9 eV, it was possible to estimate the time constant of the hole separation process that occurs in the Blend when only the A is excited, as reported in Figure 5b. The dynamics were fit by a rising exponential equation convoluted with a Gaussian function simulating the IRF and a step function (see Section S3 in Supporting Information for further details), resulting in a time constant of (1.3 ± 0.3) ps. This result is in excellent agreement with the hole separation time constant observed in similar blend systems, showing similar energy offsets.^[^ ^40^ ^]^ Moreover, this PB signal shows a progressive broadening with the time delay, which can be ascribed to the incremental population of the quasi‐free charge‐separated band.^[^ ^7^ ^]^ The ultrafast charge transfer dynamics observed through transient absorption spectroscopy indicate efficient exciton diffusion and dissociation at well‐organized donor‐acceptor interfaces. This behavior is consistent with the morphology reported in the literature in similar blends, where moderate domain sizes (≈38 nm) have been shown to facilitate effective charge separation.^[^ ^36^ ^]^

The interpretation of FTAS results closely agrees with the theoretical result obtained on a FOIC/PCE10 heterojunction and shown in Figure S1 (Supporting Information) (see the Experimental Section for calculation details). In the ground state of an interacting dyad formed by FOIC and a PCE10(4) tetramer, the HOMO is clearly localized on the polymer, while the LUMO is predominantly on the molecule (see the diagram of FOIC+PCE10(4) in Figure S1, Supporting Information). When this system is excited, its lowest energy state, S1, as described by TDDFT is a charge‐separated state, with the electron localized on FOIC and the hole on the PCE10 tetramer. This is shown in Figure 5c, where the density difference between the ground state and the charge‐separated state is represented, confirming the depletion of charge from PCE10 (blue regions) and the accumulation of charge on FOIC (red regions) upon excitation. The energy of this charge‐separated state is 1.06 eV above the ground state, in agreement with the 1.03 eV offset between the valence and conduction bands estimated on the basis of measurements.

Based on the steady‐state and TA results, the CS yield η was estimated thanks to the following equation:

(2) $$\eta =\frac{PB\left(1.90eV,20ps\right)/A\left(1.90eV\right)}{PB\left(1.45eV,t_{0}\right)/A\left(1.45eV\right)}\;$$

where *PB*(1.45 eV, *t*_0_) is the maximum value of the PB signal of the Blend at 1.45 eV probe energy, *PB*(1.90 eV, 20 ps) is the intensity of the PB signal at 1.90 eV probe energy correlated to the separated charges and collected at 20 ps, and *A(eV)* is the Blend absorption at the selected probe photon energy. In this way, it was possible to estimate a charge separation efficiency of the blend of ≈60%, considering the intensity of PB signals normalized by the corresponding absorptions, all of which were considered proportional to the population of excited charges.

## 3 Conclusion

Our study provides valuable insights into the optoelectronic properties and charge separation dynamics of a blend composed of FOIC and PCE10 organic semiconductor materials. By combining steady‐state and time‐resolved spectroscopic techniques with high‐level DFT calculations, we characterize these materials as thin films both individually and in their blended state. In particular, the femtosecond transient absorption spectroscopy investigation revealed a reduction in the bimolecular exciton‐exciton annihilation recombination rate in the acceptor when incorporated into the blend, compared to its pristine form. This reduction was attributed to a decreased exciton density within the acceptor, driven by the formation of an electron‐hole charge‐separated state caused by the transfer of a hole from the acceptor to the donor polymer, as also confirmed by TDDFT simulations. This process was characterized by following the temporal evolution of the photobleaching signals associated with the excited states of the donor when the acceptor was selectively excited within the blend. In this way, the time constant of the hole transfer from the acceptor to the donor was quantified, yielding a value of (1.3 ± 0.3) ps. Additionally, this study allowed the quantification of the exciton diffusion and the hole separation efficiency, offering valuable insights for the development of next‐generation photovoltaic materials with optimized interfacial engineering strategies to further improve the charge separation and device efficiency.

## 4 Experimental Section

### Materials

The samples investigated have a layered structure composed of glass/ITO/ZnO/PCE10:FOIC stacks. Indium tin oxide (ITO)‐coated glass substrates with a sheet resistance of 15 Ω/□ were purchased from VisionTek. Zinc oxide (ZnO) nanoparticles (2.5 wt.% in isopropanol) were purchased from Avantama. PCE10 (aka PTB7‐Th) (Poly[4,8‐bis(5‐(2‐ethylhexyl)thiophen‐2‐yl)benzo[1,2‐b;4,5‐b′]dithiophene‐2,6‐diyl‐alt‐(4‐(2‐ethylhexyl)‐3‐fluorothieno[3,4‐b]thiophene‐)‐2‐carboxylate‐2‐6‐diyl)]) was produced by KAUST. FOIC^[^ ^52^ ^]^ was purchased from 1 material.

Glass/ITO substrates were cleaned in an ultrasonic bath. ZnO nanoparticles were coated with a doctor blade and annealed for 30 min at 200 °C in an ambient atmosphere, followed by the active layer. PCE10, FOIC, and PCE10:FOIC were diluted in chloroform (22.25 mg mL^−1^ for the single components and the Blend 1:1) and stirred at RT overnight before being coated with a doctor blade using the following processing parameters: 60 µL of solution (stirred at RT) were injected into a 400‐µm gap between substrate and blade (both heated to 30 °C). Coating with 10 mm s^−1^ forms a wet film that dries very fast (2–3 s) due to the low boiling point of chloroform (61 °C). The thin films were subsequently annealed for 4 min at 140 °C under an inert atmosphere. The thickness of the films was estimated with a Brucker DecktatXT stylus profilometer to be 50 nm for FOIC, 200 nm for PCE10, and 105 nm for the Blend.

### Photoelectron Spectroscopy

Photoelectron Spectroscopy (PES) measurements were performed at the VUV‐Photoemission beamline (Elettra, Trieste) under ultra‐high vacuum conditions at room temperature using a Scienta R‐4000 electron analyzer. The surface of the organic films was sputtered by Ar ions with an energy of 500 eV for 5 min to remove the surface contamination. The energy scale of the spectra was referenced to as the Fermi level of the Mo metallic sample holder in electrical contact with the studied films.

### Transient Absorption Spectroscopy

The pump–probe experiments were performed by using a laser system consisting of a regenerative amplifier (Coherent Legend Elite Duo HE+), seeded by a Ti:Sa oscillator (Coherent Vitara‐T), that generates pulses at 800 nm (1 kHz, 4 mJ) with 35 fs of duration. The pump pulse was produced by an Optical Parametric Amplifier (OPA) at selected photon energies. Conversely, the probe beam was generated by focusing 100 µJ of the regenerative amplifier output at 800 nm into a homemade OPA that generates an output beam at 1200 nm. This femtosecond radiation is focused on a sapphire crystal to generate a white light supercontinuum probe in the VIS‐IR (500–1000 nm). The optical layout of the commercial transient absorption spectrometer (FemtoFrame II, IB Photonics) consisted of a split beam configuration in which 50% of the white light passes through the sample while the remainder was used as a reference to account for pulse‐to‐pulse fluctuations in the white light generation. The pump and the probe beams were focused on the sample with a diameter of 200 and 150 µm, respectively, and the delay time between the two was changed by modifying the optical path of the probe, resulting in an instrument response function of ≈50 fs. All experiments were performed with linear polarization for both pump (vertical) and probe (horizontal) pulses. The pump scattering is removed by means of a linear polarizer located after the sample. More experimental details on the setup can be found elsewhere.^[^ ^43^, ^53^ ^]^

### DFT

The properties of FOIC, PCE10, and their blends were investigated using a multilevel computational protocol. A preliminary broad screening of molecular configurations has been performed for each system using a conformers‐rotamers ensemble search tool (CREST^[^ ^54^ ^]^) based on the GFN‐FF force field,^[^ ^55^ ^]^ which performs a complex combination of (meta)dynamics simulations and geometry optimizations, blended by genetic sorting and mixing of structures. All the systems are isolated but immersed in a low‐polarity implicit dielectric environment to mimic the embedding into an organic film. A large ensemble of low‐energy configurations (up to hundreds of systems) is then fully reoptimized and sorted in energy using a semiempirical tight‐binding method rooted in the GFN2‐xTB Hamiltonian, as implemented in the xTB suite of programs.^[^ ^56^ ^]^ The lowest‐energy structures were then investigated using (time‐dependent) DFT‐based simulations, carefully balanced between accuracy and feasibility as applied to large systems up to 2720 electrons. Geometry optimizations were performed in a plane‐wave/pseudopotential framework using the Quantum ESPRESSO suite of programs.^[^ ^57^ ^]^ All the systems are optimized in very large supercells in periodic boundary conditions using the C09^[^ ^58^ ^]^ gradient corrected functional coupled with an ab initio VDW‐DF^[^ ^59^, ^60^ ^]^ correction (C09+VDWDF). The C09+VDWDF method provides a reliable description of structures and interaction energies of noncovalent systems. The Kohn‐Sham orbitals are expanded on a plane‐wave basis set, with core electrons replaced by ultrasoft pseudopotentials.^[^ ^61^ ^]^ Satisfactorily converged results have been obtained using 40 Ry (320 Ry) cutoffs on the plane waves (electronic densities). Accurate electronic and optical properties are then calculated on the obtained structures using a different DFT framework based on all‐electron Gaussian‐type basis sets as implemented in the ORCA suite of programs.^[^ ^62^ ^]^ Single‐point calculations were performed using the dispersion‐corrected^[^ ^63^ ^]^ B3LYP functional^[^ ^64^ ^]^ and the def2‐TZVPP all‐electron basis set.^[^ ^65^, ^66^ ^]^ The corresponding def2/J basis has also been used as an auxiliary basis set for Coulomb fitting in a resolution‐of‐identity/chain‐of‐spheres (RIJCOSX) framework. Absorption spectra were also calculated on C09+VDWDF geometries in the sTDDFT framework^[^ ^67^ ^]^ using the same B3LYP functional and the same basis sets for individual components and for a FOIC/PCE10 diad in both trimer and tetramer cases. An infinite periodic PCE10 polymer chain has been used as a reference to ensure the convergence of the electronic level of isolated oligomers. This calculation has been performed in the plane‐wave/pseudopotential framework discussed above using the same B3LYP functional. Optimized norm‐conserving Vanderbilt pseudopotentials^[^ ^68^ ^]^ have been used in this case to replace core electrons with 80/320 Ry cutoffs. For further details, see Section S1 of the Supporting Information.

## Conflict of Interest

The authors declare no conflict of interest.

## Supporting information



Supporting Information

## Data Availability

The data that support the findings of this study are available from the corresponding author upon reasonable request.
