# Supplementary material for: Electron‐Hole Separation Dynamics and Optoelectronic Properties of a PCE10:FOIC Blend
Source: Small. 2025 Jul 2;21(34):2505063. doi: 10.1002/smll.202505063 (PMC12393030; doi:10.1002/smll.202505063)
Supplement: Supplementary file 1 — Supporting Information [file SMLL-21-2505063-s001.docx]

Supporting Information

Electron-Hole Separation Dynamics and Optoelectronic Properties of PCE10:FOIC Blend

G. Ammirati^1,*^, S. Turchini^1^, F. Toschi^1^, P. O’Keeffe^1,2^, A. Paladini^1,2^, G. Mattioli^2^, P. Moras^3^, P. M*.* Sheverdyaeva^3^, V. Milotti^3^, C. J. Brabec^4,5^, M. Wagner^4,5^, I. McCulloch^6,7^, A. Di Carlo^1,8^, and D. Catone^1^.

*^1^CNR-Istituto di Struttura della Materia (CNR-ISM), EuroFEL Support Laboratory (EFSL), Via del Fosso del Cavaliere 100, 00133, Rome, Italy.*

*^2^CNR-Istituto di Struttura della Materia (CNR-ISM), 00015, Monterotondo Scalo, Italy.*

*^3^CNR-Istituto di Struttura della Materia (CNR-ISM), SS 14, Km 163.5, I-34149, Trieste, Italy.*

*^4^Forschungszentrum Jülich GmbH, Helmholtz Institute Erlangen-Nürnberg for Renewable Energy (HI ERN), Dept. of High Throughput Methods in Photovoltaics, Erlangen, Germany.*

*^5^Friedrich-Alexander-Universität Erlangen-Nürnberg, Materials for Electronics and Energy Technology (i-MEET), Erlangen, Germany*

*^6^Andlinger Center for Energy and the Environment, and Department of Electrical and Computer Engineering, Princeton University, Princeton, NJ, 08544, USA*

*^7^Department of Chemistry, Oxford University, Chemistry Research Laboratory, Oxford OX1 3TA, U.K.*

*^8^CHOSE, University of Rome “Tor Vergata”, Rome, 00133 Italy.*

**Keywords**: organic photovoltaic, charge dynamics, electron-hole separation, band diagram, photovoltaics

*Corresponding author: giuseppe.ammirati@cnr.it

## S1: Theoretical band diagram of FOIC, PCE10, and SC

On the basis of the theoretical results obtained inherent to the HOMO and LUMO levels, **Figure S1** shows the theoretical band diagram from FOIC, PCE10 (trimer, tetramer, and polymer obtained by periodic boundary conditions, labeled respectively as (3), (4), and (PBC)) and the FOIC+PCE10 (4) obtained with the B3LYP functional. The theoretical evaluation demonstrates that, concerning the energetics of frontier orbitals, even a tetramer is essentially converging with the PBC system, while a trimer is only partially satisfactory; for this reason, the calculation were performed on the FOIC:PCE10 (4) diad to ensure more reliable results.


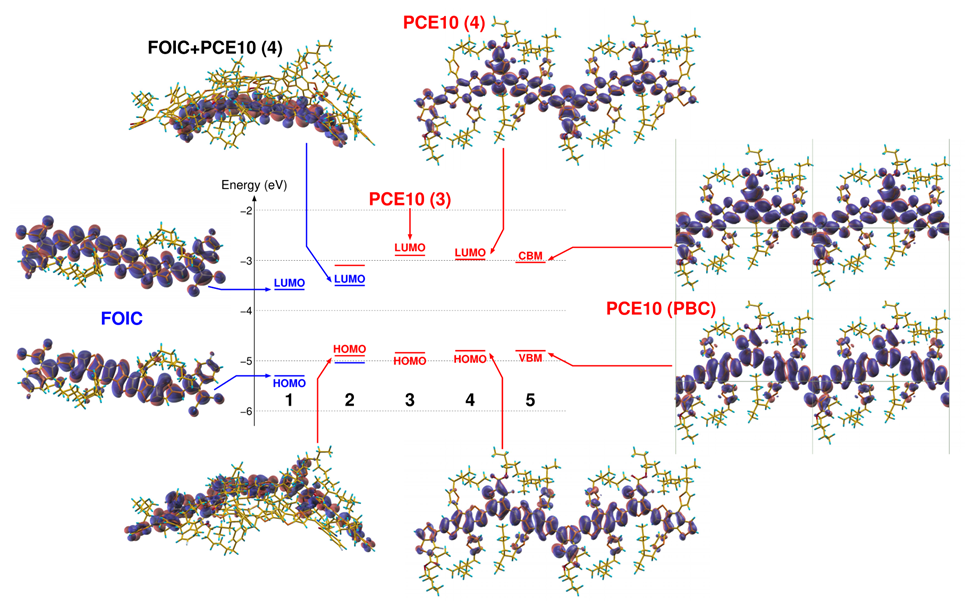


**Figure S1:** Theoretical band diagram for FOIC, PCE10 trimer (3), tetramer (4), and infinite crystal (PBC), and the PCE10(4): FOIC Blend.

Absorption spectra were also calculated on C09+VDWDF geometries in the sTDDFT framework^32^ using the same B3LYP functional and the same basis sets for individual components and for a FOIC/PCE10 dyad in both trimer and tetramer cases. The simulations quite accurately replicate the spectroscopic features of the molecule, notably the strong near-infrared (NIR) absorption peaking at 869 nm (1.43 eV), while for PCE10 the pronounced absorption measured between red and NIR wavelengths shifts to lower energies with respect to the experimental absorption maximum (1.72 eV): the absorption peak is at 1.51 eV for the trimer and 1.39 eV for the tetramer. **Table S1** reports DFT calculation with B3LYP functional of the HOMO and LUMO energy levels and absorption peak energies. The theoretical data reported in the main text, in **Table S1** highlighted in blue, are FOIC, PCE10 (4) and FOIC+PCE10 (4) structures, as they result to be closer to the experimental results.

|  | HOMO (eV) | LUMO (eV) | Absorption peak (eV) |
| --- | --- | --- | --- |
| FOIC | -5.31 | -3.58 | 1.50 |
| PCE10 (PBC) | -4.80 | -3.06 |  |
| PCE10 (3) | -4.84 | -2.91 | 1.51 |
| PCE10 (4) | -4.80 | -2.98 | 1.39 |
| FOIC+PCE10 (3) | -4.89 | -3.48 | 1.61 |
| FOIC+PCE10 (4) | -4.89 | -3.50 | 1.46 |

**Table S1:** DFT calculation with B3LYP functional of the HOMO and LUMO energy levels and the theoretical absorption peak energies calculated on C09+VDWDF geometries in the sTDDFT framework.

## S2: Experimental photoelectron spectra and estimation of ionization energies of FOIC, PCE10, and Blend

The onset of the Valence Band (VB) of each material was defined as the intersection between the a linear fitting of the VB spectrum in the low binding energy region and a baseline, corresponding to the signal above the Fermi energy (dark counts) . Since the very broad experimental spectra do not allow to determine a unique onset energy, the straight line was fitted by varying the constrained axis limit between 0.7 and 3 eV with a fixed energy window of 500 meV. **Table S2** reports the mean value and the standard variation of binding energy onset of the VB, while **Figure S2** reports some selected fitting curves obtained with the previously described method.

On the basis of the experimental photoelectron spectroscopy (PES) and absorption spectra, the VB maximum and conduction band (CB) minimum was estimated as follows:

| $\vert VB\vert=\vert\Phi\vert+\vert E_{b}\vert$  $\left\vert CB \right\vert=\left\vert VB \right\vert-E_{g}$ | S1 |
| --- | --- |

where Φ is the work function of the analyzer (4.51 eV), E_b_ is the PES binding energy onset and E_g_ is the optical bandgap onset.


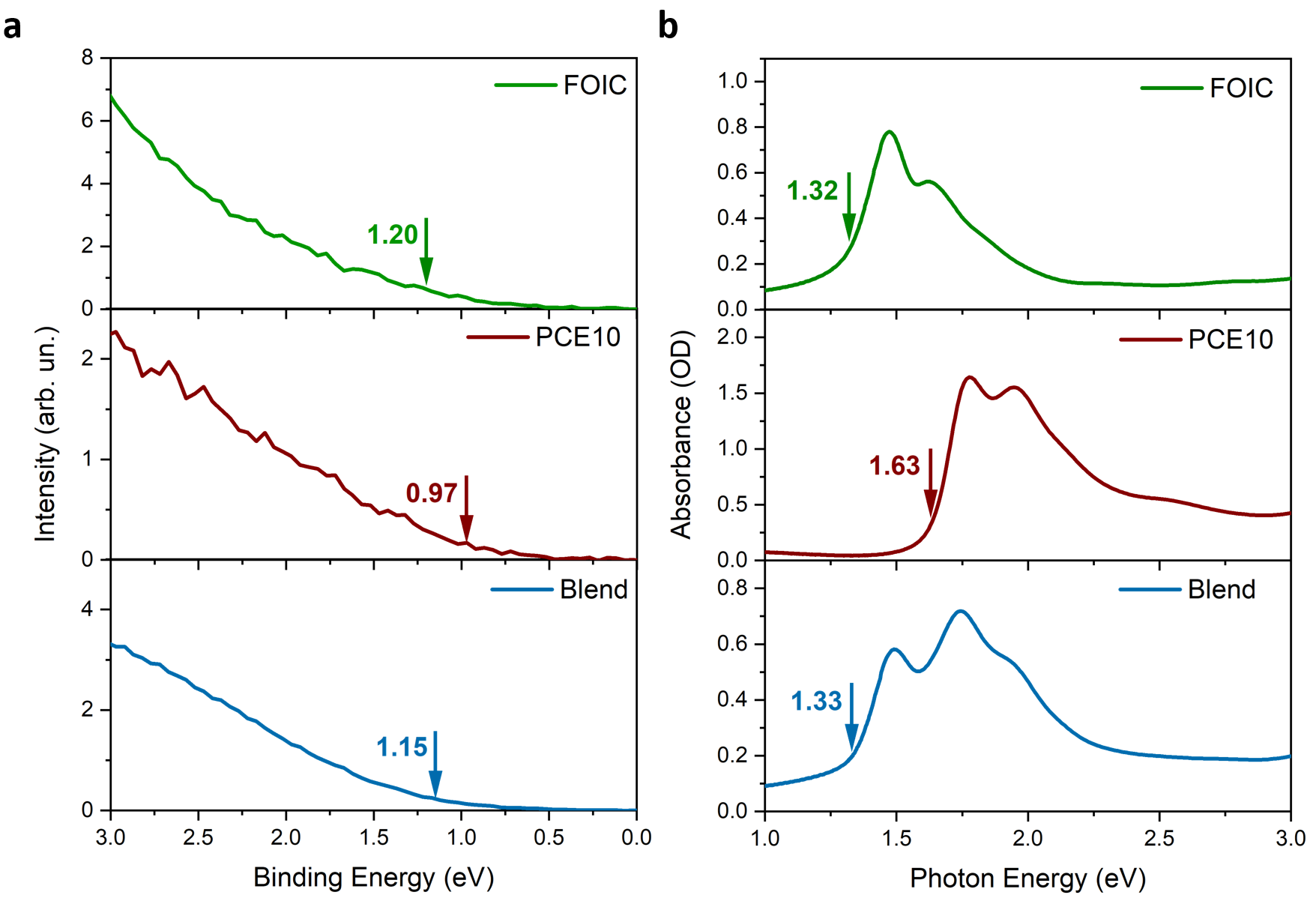


**Figure S2:** Experimental (a) PES spectra acquired at the photon energy of 75 eV and (b) absorption spectra obtained of the film of FOIC (green line), PCE10 (red line), and Blend (blue line).

All the values estimated using this method are reported in **Table S2**.

|  | FOIC | Blend | PCE10 |
| --- | --- | --- | --- |
| E_b_ (eV) | 1.20 | 1.15 | 0.97 |
| E_g,_ (eV) | 1.32 | 1.33 | 1.63 |
| VB (eV) | -5.71 | -5.66 | -5.48 |
| CB (eV) | -4.39 | -4.33 | -3.85 |

**Table S2:** Experimentally estimated E_b_ and E_g_, and the calculated VB maximum and CB minimum, for the FOIC, PCE10, and Blend.

## S3: Transient absorption spectroscopy

The exciton density generated by the pump excitation (n_0_) is defined as follows:

$$n_{0}=\frac{F}{\hbar\omega}\cdot\frac{A}{d}$$

Where F is the pump fluence, ћω is the pump photon energy, A is the absorptance of the sample (i.e., the fraction of absorbed photons, see **Figure S3**), and d is the sample thickness. The thickness was estimated to be 50 nm for FOIC; 200 nm for PCE10; 105 nm for Blend. The values used for the estimation of the exciton density are reported in **Table S3**.

| Materials | Pump Energy (eV) | Absorptance (%) | Thickness (nm) |
| --- | --- | --- | --- |
| FOIC | 1.45 | 39 | 50 |
| Blend | 1.45 | 33 | 105 |
| PCE10 | 1.75 | 70 | 200 |

**Table S3**: Experimental values used for the estimation of the exciton density at the experimental pump photon energy and fluence.


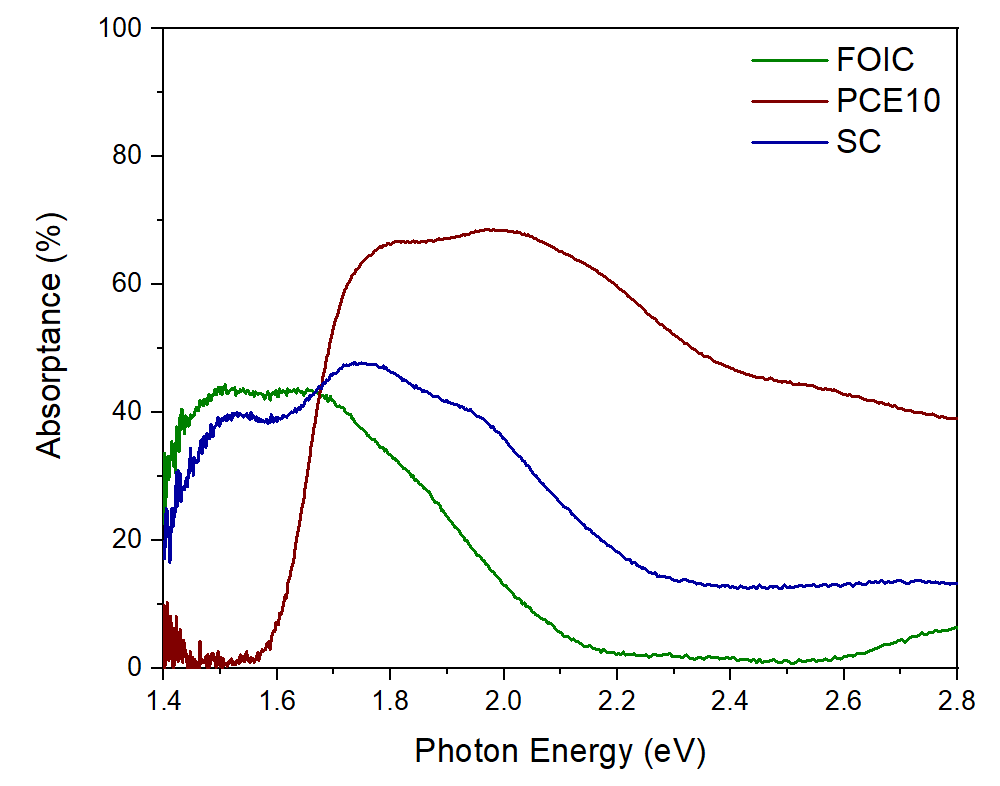


**Figure S3:** Absorptance spectra of FOIC (green line), PCE10 (red line), and Blend (blue line) obtained by the UV-2600 Shimadzu spectrometer with the multipurpose sample compartment add-on.

### S3.1 Fitting of the temporal dynamics

The fitting equations used to fit the exciton-exciton in this work is the following.

| $\Delta A\left( t \right)=\frac{\Delta A_{2}}{1+\frac{t-t_{0}}{\tau}}+\Delta A_{0}$ | S2 |
| --- | --- |

The bimolecular decay time τ extracted from the curve fitting is directly correlated to the bimolecular recombination rate γ by the following formula:

| $\frac{1}{\tau}={\gamma\cdot n}_{0}$ | S3 |
| --- | --- |

where n_0_ is the exciton density.

The estimated carrier density and time constants estimated by this curve fitting and reported in **Figure 4** in the main text is reported in the following **Table S4:**

| FOIC | |
| --- | --- |
| Carrier Density (10^19^ cm^-3^) | τ (ps) |
| 1.8 | 2.2±0.4 |
| 4.4 | 1.8±0.2 |
| 6.3 | 1.15±0.09 |
| 19 | 0.46±0.02 |
| 31 | 0.37±0.02 |
| 44 | 0.28±0.02 |
| 63 | 0.17±0.03 |
| Blend | |
| Carrier Density (10^19^ cm^-3^) | τ (ps) |
| 2.4 | 3.5±0.4 |
| 5.6 | 2.4±0.3 |
| 8.0 | 2.0±0.2 |
| 24 | 0.74±0.08 |
| 40 | 0.65±0.12 |
| 56 | 0.35±0.06 |
| 80 | 0.7±0.2 |
| PCE10 | |
| Carrier Density (10^19^ cm^-3^) | τ (ps) |
| 0.83 | 51±5 |
| 2.0 | 27±2 |
| 2.8 | 13.5±1.5 |
| 8.4 | 8.8±1.1 |
| 14 | 4.8±0.7 |
| 20 | 2.6±0.3 |
| 28 | 2.2±0.2 |

**Table S4**: EEA decay time obtained for FOIC, PCE10, and Blend at different excited carrier densities.

The rise time of the PB signal was fitted with the following formula:

| $\Delta A_{rs}\left( t \right)=A_{1}\cdot\left[ \left( 1+erf\left( \frac{\left( t-c \right)}{\sqrt{2}\sigma} \right) \right)-e^{\frac{\sigma^{2}}{2\tau_{2}^{2}}-\frac{\left( c+t \right)}{\tau_{2}}}\left( 1+erf\left( \frac{t-c}{\sqrt{2}\sigma}-\frac{\sigma}{\sqrt{2}\tau_{2}} \right) \right) \right]+A_{o}$ | S4 |
| --- | --- |

where $\Delta A_{rs}\left( t \right)$ is the rising temporal trend of the transient signal collected at selected probe energy, $A_{1}$ is the amplitude of the transient signal, $c$ is the time where the maximum of the laser pulse takes place, the temporal form of the pulse is described by a Gaussian with a standard deviation of $\sigma\approx0.03ps$, t is the time delay between pump and probe, $\tau_{2}$ is the rise time of the transient signal and *A_o_* is the offset which describes the mean transient intensity value at negative times.

### S3.2 FTAS results at the pump energy of 1.72 eV

**Figure S4a** shows the measured TA spectra obtained with a pump photon energy of 1.72 eV and at a fluence of 220 µJ/cm^2^ for FOIC, PCE10, and Blend. The TA spectra show PB signals where the absorption spectra show intense peaks. In fact, FOIC shows an intense PB signal at 1.45 eV and PCE10 at 1.72 eV. Both systems show also negative features at higher energies that were attributed to the vibrational state progressions already discussed in the main text. On the other hand, the Blend exhibits feature that can be readily identified with those found in the TA spectra of the individual components: a strong PB signal at 1.72 eV, associated with a donor-like transition; a less intense PB signal at 1.45 eV, associated with an acceptor-like transition. The different intensity of the PB signals since the energy of the pump (1.72 eV) is almost resonant with the donor-like optical transition, inducing a more substantial change in the electronic occupation of the states involved in the excitation.

**Figure S4b** shows the temporal dynamics obtained at the pump photon energy of 1.75 eV with a fluence of 220 µJ/cm^2^ and at the probe photon energy of 1.72 eV for PCE10 and of 1.45 eV for FOIC, namely at the energies of the PB signal minima.


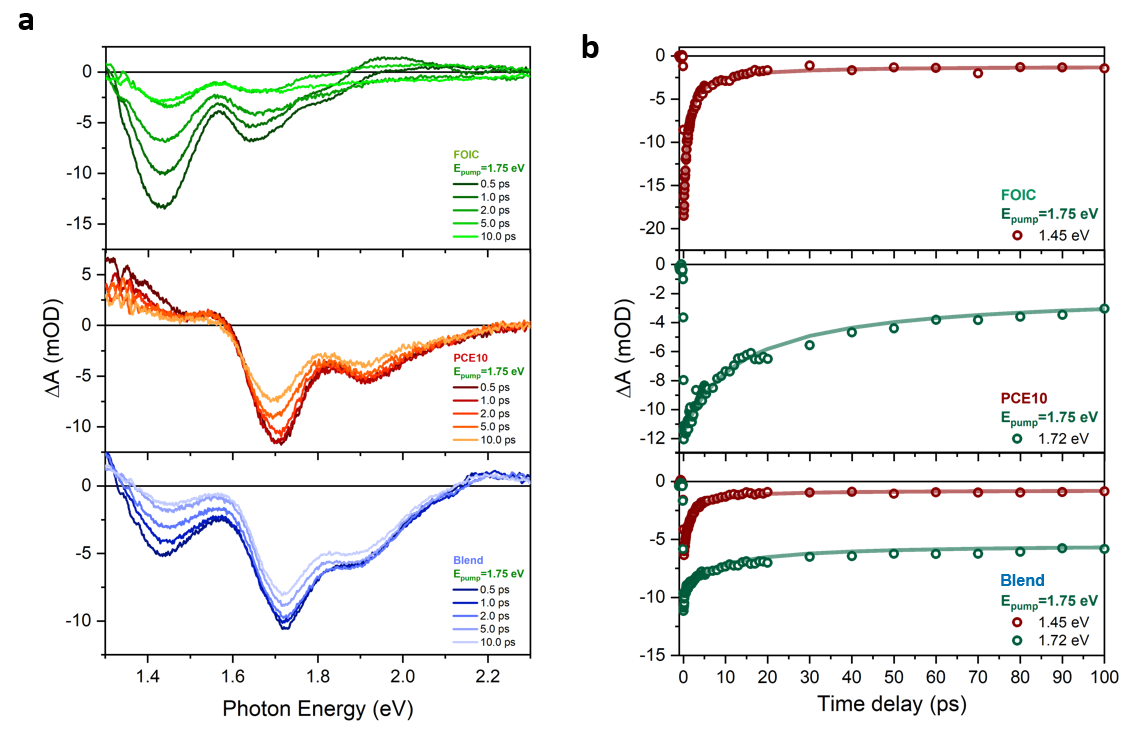


**Figure S4:** (a) TA spectra were obtained at the pump fluence of 220 µJ/cm2 and the pump photon energy of 1.75 eV for PCE10 (red), FOIC (green) and Blend (blue); (b) temporal dynamics obtained at the pump fluence of 220 µJ/cm^2^ and the pump photon energy of 1.75 eV for PCE10 (green scatter the temporal dynamics at the probe photon energy of 1.72 eV), FOIC (red scatter the cuts at the probe photon energy of 1.45 eV), and Blend (red and green scatter the cuts at the probe photon energy of 1.45 eV and 1.72 eV respectively).

The Blend was studied in the same excitation conditions and the temporal dynamics were explored at the probe energies selected for PCE10 and FOIC. The experimental results demonstrates that the decay of the PB signals lasts for tens of picoseconds, in agreement with the observed trend in other organic thin films^18–20^ and their decay was attributed to EEA process. The estimated time decay for FOIC, PCE10, and Blend are reported in **Table S5.**

|  | FOIC | Blend | PCE10 | Blend |
| --- | --- | --- | --- | --- |
| Probe photon energy (eV) | 1.45 | 1.45 | 1.72 | 1.72 |
| τ(ps) | 0.92±0.05 | 1.12±0.07 | 13.5±1.5 | 6.9±1.1 |

**Table S5**: EEA decay time obtained for FOIC, PCE10, and Blend at the pump photon energy of 1.75 eV obtained at the different probe photon energy.

The decay times obtained by the fit procedure show that FOIC exhibits a faster decay with respect to PCE10. Additionally, the Blend shows similar trends but not identical to those recorded from the pristine FOIC and PCE10. This behavior is ascribable to the fact that under this experimental condition both the donor and acceptor are excited in the Blend, making it difficult to disentangle the effect of the excitation of the single component.

To gain a clearer understanding of the recombination mechanisms underlying the different temporal dynamics, FTAS measurements were performed on FOIC and Blend at a pump photon energy of 1.45 eV. In this way, only the acceptor in the Blend was excited, giving the opportunity to follow the charge dynamics of a single component of the Blend (see the main text).
